# Hospital and Institutional News

**Published:** 1919-07-19

**Authors:** 


### HOSPITAL AND INSTITUTIONAL NEWS.

### THE AMERICAN HOSPITAL IN LONDON.

French, Italian, and German Hospitals, London has long known, and no more fitting mark of the close sympathy now happily existing between the two main branches of the Anglo-Saxon stock could be imagined than the addition to the three mentioned of an American Hospital. A project for such an institution has been on foot for some time, and is now assured of fruition. The prime mover is an American surgeon, Mr. Philip Franklin, and the other members of the (London) Executive Committee are Sir William Osler, Sir Arbuthnot Lane, Sir Humphry Rolleston, and Sir John Bland Sutton. Sir Arbuthnot Lane has recently expounded the project, which has important educational ideals as well as purely therapeutic ones, to the congress of the American Surgical and American Medical Associations held recently in Atlantic City. To establish American co-operation upon a definite footing, a committee was formed to act with the Executive Committee in London, and, if desirable, to operate under the National Research Council at Washington. This American Committee comprises Dr. G. W. Crile, Drs. W. J. and C. H. Mayo, Dr. A. J. Ochsner, Dr. Rudolph Matas, and Dr. Franklin Martin—as distinguished a band, it may be remarked, as the American profession could possibly select. The hospital has already been selected as the meeting-place of the newly formed American Post-Graduate Society for London, and it appears probable that research will form no small part of its activities. As a form of a War memorial fitly commemorating Anglo-American comradeship on the battlefield, the appeal of this new institution will obviously be powerful, and The Hospital wishes the new venture and its managers nothing but success. No statement has yet appeared as to the probable locality of the new institution, but a meeting of Committees is, we understand, being held as we go to press. Possibly King Edward VII. Fund could give valuable help in deciding this point.

### THE HOSPITAL AT PESHAWAR.

Our recent article on "The Afghan War and the Khyber Pass," describing the medical mission work in the frontier hospitals has brought us some further information concerning the C.M.S. Hospital at Peshawar. After the murder of Dr. Vernon Starr in March 1918 Dr. Cox become the head of the hospital. We now learn that in May he and his staff joined the Indian General Hospital, and have received as many as 500 cases in one day. The staff and equipment have been voluntarily placed at the disposal of the authorities, and have been warmly welcomed because the Indian Medical Service is very short-handed. Details of the work on the frontier may be expected from the biography of Dr. V. H. Starr, which we hope to have an opportunity to review as soon as it appears. It will be published privately.

### £10,000 FOR THE LEAGUE OF MERCY.

Sir Frederick Green, K.B.E., hon. treasurer of the League of Mercy, has presented £10,000 to the League, "in the hope that now that H.R.H. the Prince of Wales has consented to become Grand President a new era of activity of the League may be inaugurated," and that "this may be allowed to form a nucleus for further donations, the investment of which should help to secure, in the future, more stability and continuity than have hitherto been possible." While recognising Sir Frederick Green's generosity, we feel bound to point out that the League of Mercy was established by King Edward to raise £50,000 a year in small subscriptions of 10*s*. and under. The experience in the case of the Sunday Fund, to which the late George Herring left a sum which has produced in income as much as £35,000 in one year, has been that the receipt of a great gift tends largely to reduce the amounts received from the congregations. These fell from nearly £50,000 a year in 1903 to as little as £28,400 in 1913, whereas the number of contributing congregations in the former year was 2,013 only, and increased to 2,125 in 1913. With the exception of last year the yield of the congregations has been well below £40,000 a year. The improvement in the yield in 1918 was due to a new system of collection introduced in the last two years with encouraging success. Legacies have always been welcomed by the Sunday Fund, but a great gift of money is calculated to prove injurious to the yield in revenue from large numbers of small contributors, and we fear large gifts to the League of Mercy, however generous and well intentioned, must tend to have the same effect on this purely collecting agency. We may add that we are fully conscious that it is possible to raise at least £50,000 a year through the League of Mercy, providing it is organised mainly or, as we are convinced, entirely on the principle of personal service, voluntarily and cheerfully rendered in the days of health in the cause of the sick. This principle on which the League was created and worked in its earliest days will, we hope, be again put forward and maintained as the principle on which the League is to be administered for the future. It will then do honour to the memory of our beloved King Edward, and should prove the means of rapidly and adequately raising the revenue for the hospitals from this source in future years. Personal service is a vital principle, very precious to all who have learnt to practice it through the institution of the League of Mercy, which should be kept absolutely free from all adventitious aids in connection with the raising and the harvesting of its income for each year.

### PHYSICAL EDUCATION.

Major-General Wood, of the U.S. Army, in a recent article emphasises the importance of physical education and training from early childhood. He states that approximately thirty per cent. were rejected by medical boards as unfit for military service. Many of the physical defects were of a character which could have been easily corrected in early youth through proper training and exercise. He calls for much broader and deeper interest on the part of the State in the welfare of its youth, the establishment of measures looking to the building up of physical training and general sanitary supervision in public schools and in all places where the youth come under the supervision and control of the State. Carelessness concerning the person brought out very forcibly the necessity of more careful teaching in matters that have to do with personal hygiene. The figure given above is worth noting when it comes from a nation of such relative youth and rapid growth as America, and may be compared with the far lower A1 percentage experienced by medical boards in this more solely pressed country.

### EARLY VISITATION OF CONSUMPTIVES.

Professor Henry Kenwood points out in his advice to the Stoke Newington Council, that in the campaign against tuberculosis it is of primary importance to provide for visitations to the homes of sufferers by a skilled person, such as the tuberculosis officer, in order to get hold of cases earlier than is otherwise possible. Only thus, he remarks, can we hope to reduce as far as practicable the present large expenditure of time and money upon cases in which the disease is so far advanced that little more than a temporary ' patching up ' can be hoped for. Unless more treatment can be applied at the outset, it is a question whether the present benefits to the community are commensurate with the heavy cost of obtaining them. The situation, therefore, demands that the tuberculosis medical officer must keep in the closest possible contact with the general practitioners and the homes.

### "TEN-MINUTE CURES."

With very commendable emphasis and with dignified restraint the *Times* has condemned in a leading article an official statement sent out recently by the Ministry of Pensions. This most unfortunate announcement alluded to the "ten-minute cures of disabled men by psycho-therapy at Seale Hayne Military Hospital, Newton Abbot.'" As the *Times* rightly points out, such a prospect, thus held out officially, will only result in cruel disappointment for numbers of disabled soldiers, who do not appreciate that these rapid cures are only possible for cases of war hysteria, which have been not uncommon. Further, this treatment has been carried out in scores of military hospitals other than Seale Hayne, which latter is, indeed (as it now appears), actually closed down. Altogether the Pensions Ministry has made a bad blunder over this question, a blunder incomprehensible in its stupidity. What irritates the public, especially when Government departments make such silly "howlers," is the uncertainty whether the responsible individual is duly reprimanded, or whether he may not actually obtain promotion or a decoration if only he wirepulls with sufficient skill and pertinacity.

### CHILD EMPLOYMENT.

Dr. Burpitt, in his report to the Newport (Mon.) Education Committtee, devotes several pages to a discussion of the effect of employment of school children out of school hours. Summarising his conclusions, Dr. Burpitt tells us that the body reacts on the mind, and children who work too long, or too hard, are too tired to get the full benefit of their education, and are unfairly handicapped in their competition with those who do not work. Physically, although it is not so easy to show ill-effects of excessive work on a large scale, yet a considerable undermining of the vitality and preparation for future breakdown occurs; and those in constant contact, such as the teachers, are able to give many instances of children who have suffered impairment of health since commencing employment. It is, therefore, essential that every employed child should be under supervision, to ascertain that he is placed in work for which he is fitted, and that the conditions do not become too onerous. In many parts of the country this is a matter which calls for considerable reforms.

### TRADES GENEROSITY.

The butchers who belonged to the Islington section of the Union of London Retail Meat Trades, and gave a motor-ambulance to the 7th (Highgate) Section of the V.A.D., have now presented it to the Great Northern Central Hospital. Mr. Frank Hammond, after making the presentation, promised to invite the Islington butchers to subscribe to the local War Memorial, which is to take the form of an extension of the Great Northern Hospital. The drapers of the district have already given £800 for this purpose, and it is hoped that the different trades will vie with each other to raise the highest amount.

### LIVINGSTONE COLLEGE, LEYTON.

It will be of interest to our readers to learn that Dr. T. Jays, whose work with the Church Missionary Society is well known, has accepted the post of Vice-Principal at Livingstone College, where the sessions will commence on October 1. Dr. Jays and his wife will be resident in the College, and in addition to helping the Principal, Dr. Wigram, in other ways, will take the lectures previously delivered by Colonel G. B. Price. The latter has gained the well-merited honour of C.M.G. for his work in Malta during the war, and is now taking up important work under the Ministry of Pensions to organise and advise on the treatment of discharged soldiers suffering from tropical diseases. Before the College reopens, a short course of fifteen lectures on Personal Care of Health in the Tropics will be given from September 22-25; Colonel Price hopes to be able to take some of these lectures, the others will be given by the Principal and Vice-Principal. Application should be made previous to the lectures, to the Principal, Livingstone College, Leyton, E. 10, who will be pleased to answer any questions with reference to these lectures, or concerning the full nine months' course at Livingstone College, which will commence on Wednesday, October 1, 1919.

### DECORATIONS AND FIRE RISKS.

When we are decorating and illuminating our hospitals with the breezy bunting and the flickering fairy lamp we must keep well in view the extra fire-risk caused by decorations. The British Fire Prevention Committee, who look so well after our welfare through their system of warnings at timely seasons, point out in a special circular that fires at holiday time and on occasion of public rejoicing usually occur in buildings filled with people, and may become holocausts. They urge the importance of dangers, especially in crowds, through smoking, and recommend that all bunting and flags should be kept well clear of open lights and fires, and away from electric switchboards and fuses. Celluloid, being a highly inflammable material, should not be used in decorative schemes, and tissue paper (unless properly treated) should not be used as a decoration or covering for illuminated globes. Apart from the most important question—risk to life in a conflagration—it must be remembered that a building that has been burnt down in these times is not quickly replaced, and the cost of rebuilding would probably be 130 per cent. more than the original cost. Many hospital managers will be much easier when the fireworks have finished and the decorations departed.

### KING EDWARD VII.'S HOSPITAL, CARDIFF.

The July meeting of the Board of Management of King Edward VII. Hospital, Cardiff, was the first held since the deaths of Colonel Bruce Vaughan and Mr. John Morgan, the Chairman of the Finance Committee. Remarkable tributes were paid to Colonel Bruce Vaughan's unique work for the hospital, General Lee describing him as the mainspring of the work at the institution. Of Mr. Morgan the General said: "He was a very sturdy character, whose great moral courage had helped them very much." Many members joined in the general appreciation, including Major Paterson, the Chairman of the Medical Board, and a large number of colliery and other working-men representatives, who have always regarded Colonel Vaughan's devotion as an inspiration to their own efforts to help the hospital. The meeting revealed a united determination to persevere in the work to which the Colonel's life had been consecrated, and indications were not wanting that a memorial would be initiated with the object of completing the extensions to which he was committed. There should be no unnecessary delay in promoting a memorial, for experience proves that time is an important factor.

### PROPOSED PSYCHIATRIC CLINIC AT CARDIFF.

An effort is being made to promote a psychiatric clinic at Cardiff in which the Mental Hospital, which is considered to be one of the best in the Kingdom, may co-operate with King Edward VII. Hospital in conjunction with the Welsh National Medical School. It is considered that a so-called "general" hospital should provide facilities for consultation and treatment of the early stages of mental disease, so that patients suffering in this way would not be prejudiced by the stigma so commonly associated with mental trouble. In order to inform and stimulate public opinion on the matter a thoughtful contribution by Colonel Hepburn, the Dean of the Medical Faculty at Cardiff, has been published in the *Western Mail*, and that Journal has powerfully advocated in its editorial columns the need of such provision, for which Cardiff appears to supply special opportunities.

### HOSPITAL HOLD-UPS.

The two youths charged with being concerned in the armed attack on Mr. Curry, clerk to the governors of Guy's Hospital, had another appointment at Bow Street Police Court last week, where they were charged with robbing a safe at a West End restaurant, which act was also, according to the evidence, associated with violence and the use of revolvers in quite the cinematograph style. They were remanded for eight days. Another attempted hospital burglary was on Monday last described at the Marylebone Police Court when a man was charged as a suspected person found at the North-Western Fever Hospital, Lawn Road, Hampstead, and with assaulting Miss Winifred Gauntlett, a staff-nurse. The man, who had been a temporary porter at the hospital, was early on Monday morning found by the nurse standing by her bedroom door in the home. He approached her, she stated in her evidence, saying "I am a burglar," and struck her on the head with his fist. After he had again struck her, she managed to get into the corridor and screamed for help, when other nurses came to her assistance. When the magistrate ordered a remand, at the prisoner's request, he agreed to an examination by a doctor in order to ascertain the mental condition of the accused.

### THE SOUTH LONDON HOSPITAL FOR WOMEN.

Anniversary Day at the South London Hospital for Women, Clapham, was a successful function, and augurs well for the raising of £7,000, which is required to carry on the work. The Marchioness of Londonderry, who presides over the board of management, was successful in obtaining the assistance of the Marchioness of Carisbrooke, who received the purses of money collected. The amount thus raised was over £700, so that it is confidently expected that ere long the whole sum of £7,000 will have been subscribed. Last August the Queen honoured the hospital with a visit, and on this occasion it was only other pressing engagements that kept her away from the hospital, in which, as patron, she takes the liveliest interest.

### THE DECREASED OUTPUT OF COAL EXPLAINED.

At the formal opening of the Orthopædic Sub-centre at Sunderland, which is to be enlarged in connection with the extension of the Royal Infirmary, Mr. W. Handy, a miner, who is chairman of the Workmen's Governors, volunteered an explanation of the decreased output of coal. He said that the miners are not to blame. They have been underfed, and are run down because of the amount of work which they have performed during the War. Give the miners, Mr. Handy exclaimed, more food and they will produce more coal. So far as we are aware this is the first time that such an explanation has been suggested, and it will come as a surprise to the public, which has always supposed that miners, whether in Durham or Wales, fed themselves better than other classes.

### THE HOSPITAL FOR TROPICAL DISEASES.

A REUNION was held last week at Endsleigh Palace Hospital for officers of previous patients, nurses, and V.A.D.s before its original work comes to an end on the 15th. Sir Archibald Williamson, M.P., chairman of the committee, received the guests. It is four years since the hospital was opened as an auxiliary institution, supported by voluntary subscriptions. The building will now be handed over to the Seamen's Hospital Society, and will therefore be one of those not to revert to general use. The Seamen's Hospital Society will use the building for sailors and soldiers suffering from tropical diseases. It will contain fifty beds, and when the victims of the war have all been dealt with the beds will be reserved for sailors. The institution will be called the Hospital for Tropical Diseases, Endsleigh Gardens.

### JUBILEE OF AN AUSTRALIAN HOSPITAL.

A NUMBER of the pages of the current number of the *Prince Alfred Hospital Gazette* are taken up by lists of contributions to the Jubilee Fund; disproving, or proving as an exception, the generally accepted theory that when State aid steps in at the door of a hospital voluntary aid jumps out of the window. The public has supported the Jubilee appeal right royally and many gifts, such as that of £1,120 from the Australian Jockey Fund, are earmarked for desirable special improvements, such as a ward for returned tuberculous soldiers or a medical gymnastics department. Concurrently with the appeal, the hospital was called upon for considerable aid in the influenza epidemic, and thus had one more opportunity of showing its great usefulness. Of any event comparable the oldest inhabitant had no recollection, and a general hospital was for the time being converted into an infectious hospital and all but urgent cases, medical and surgical, had to give place to cases of pneumonic influenza. At one time 300 patients were being treated, and no fewer than seven resident medical officers and forty-three nurses were ill with the disease. Mr. William Epps, the Secretary of Prince Alfred Hospital and a hospital officer well-known in this country, has been appointed by the Department of Industry as a member of the new Industrial Board to deal with industrial matters connected with the staffs of hospitals and kindred institutions.

### CAPITAL FROM LABOUR.

AN extremely successful start has been made with the Ipswich War Memorial. For the new wing which is to be added to the East Suffolk Hospital a sum of £32,000 was promised before public meetings were held, and it has now been decided to hold a collection to provide a capital sum in the works, where weekly contributions are already given. The new wing will contain three wards for eye cases, ear, nose, and throat cases, and maternity cases respectively. The nurses also need better quarters than they have at present. That the proposed collection has been welcomed shows that the workmen's representatives are ready to contribute to capital as well as to maintenance, and we are glad to see that the example of the richer friends of the hospital has been followed.

### COTTAGE HOSPITALS AND SECTIONAL SUBSCRIPTIONS.

THE Colwyn Bay Cottage Hospital is in need of extension, particularly of a new children's ward, and an endeavour is being made to induce other sections of the population besides the workers in the lime quarries to organise regular contributions. It is nearly twenty-five years since this public institution was founded, but the existing buildings were designed for their purpose and there is sufficient land to enable the extensions to be carried out. Mr. J. W. Raynes, the chairman, states that the time has come for the number of beds to be doubled, and if the example of the quarrymen is followed by clerks and in the shops of the neighbourhood there is no reason why this development should not take place. We hope, too, that the employers, as well as workpeople, will be asked to give a sum in proportion to that subscribed by their employees. If the former decide on a joint plan, it is easier to arrange a really corporate effort.

### THE NEW EAST SUSSEX HOSPITAL.

"THERE is no war memorial that in utility—I might almost say in holiness—equals that of the rebuilding and enlargement of a great hospital," said Sir Rider Haggard at Hastings the other day at a gathering on the site of the new East Sussex Hospital. The plan is to remove the present hospital from the sea-front to a new site, at a cost of some £60,000; and if the enthusiasm for peace is sincere the sum should find ready donors among all, including those who are so quick to subscribe to the funds for Peace celebrations.

### THIS WEEK'S DRUG MARKET.

GENERALLY speaking there is an all-round tendency towards rather higher prices. This, however, may only be temporary. The upward tendency is to a large extent due to the increased demand for drugs in the past both of the home and overseas trade. The embargo on the importation of chemical drugs has had an unsettling effect on trade and accounts to some extent for the higher prices that are being asked for various synthetic drugs. As was expected, makers of calomel, corrosive sublimate, and other salts of mercury have advanced their quotations in consequence of the rise in the value of quicksilver. Menthol continues to attract attention and is again rather dearer; the same applies to Japanese peppermint oil. English refined camphor has again been advanced in price. Clove oil is dearer in consequence of the advance in cloves. Salicylic acid, salicylate of soda, aspirin and phenazone are dearer. Eucalyptus oil has a downward price tendency.

